# Supplementary material for: Interrogating ligand-receptor interactions using highly sensitive cellular biosensors
Source: Nat Commun. 2023 Nov 28;14:7804. doi: 10.1038/s41467-023-43589-1 (PMC10684770; doi:10.1038/s41467-023-43589-1)
Supplement: Supplementary file 1 — Supplementary Information [file 41467_2023_43589_MOESM1_ESM.pdf]

Supplementary Information

Interrogating Ligand-Receptor Interactions Using Highly Sensitive Cellular Biosensors

Maximilian A. Funk[1], Judith Leitner[1*], Marlene C. Gerner[2], Jasmin Hammerler[2], Benjamin Salzer[3,4], Manfred Lehner[3,4], Claire Battin[1], Simon Gumplmair[1], Karin Stiasny[5], Katharina Grabmeier-Pfistershammer[6], and Peter Steinberger[1*]

[1]Center for Pathophysiology, Infectiology and Immunology, Institute of Immunology, Division for Immune Receptors and T cell activation, Medical University of Vienna, Vienna, Austria
[2]Division of Biomedical Science, University of Applied Sciences FH Campus Wien, Vienna, Austria.
[3]St. Anna Children´s Cancer Research Institute, Vienna, Austria
[4]Christian Doppler Laboratory for Next Generation CAR T Cells, Vienna Austria
[5]Department of Virology, Medical University of Vienna, Vienna, Austria
[6]Department of Dermatology, Medical University of Vienna, Vienna, Austria

*e-mail:
judith.a.leitner@meduniwien.ac.at
peter.steinberger@meduniwien.ac.at

Supplementary Figures

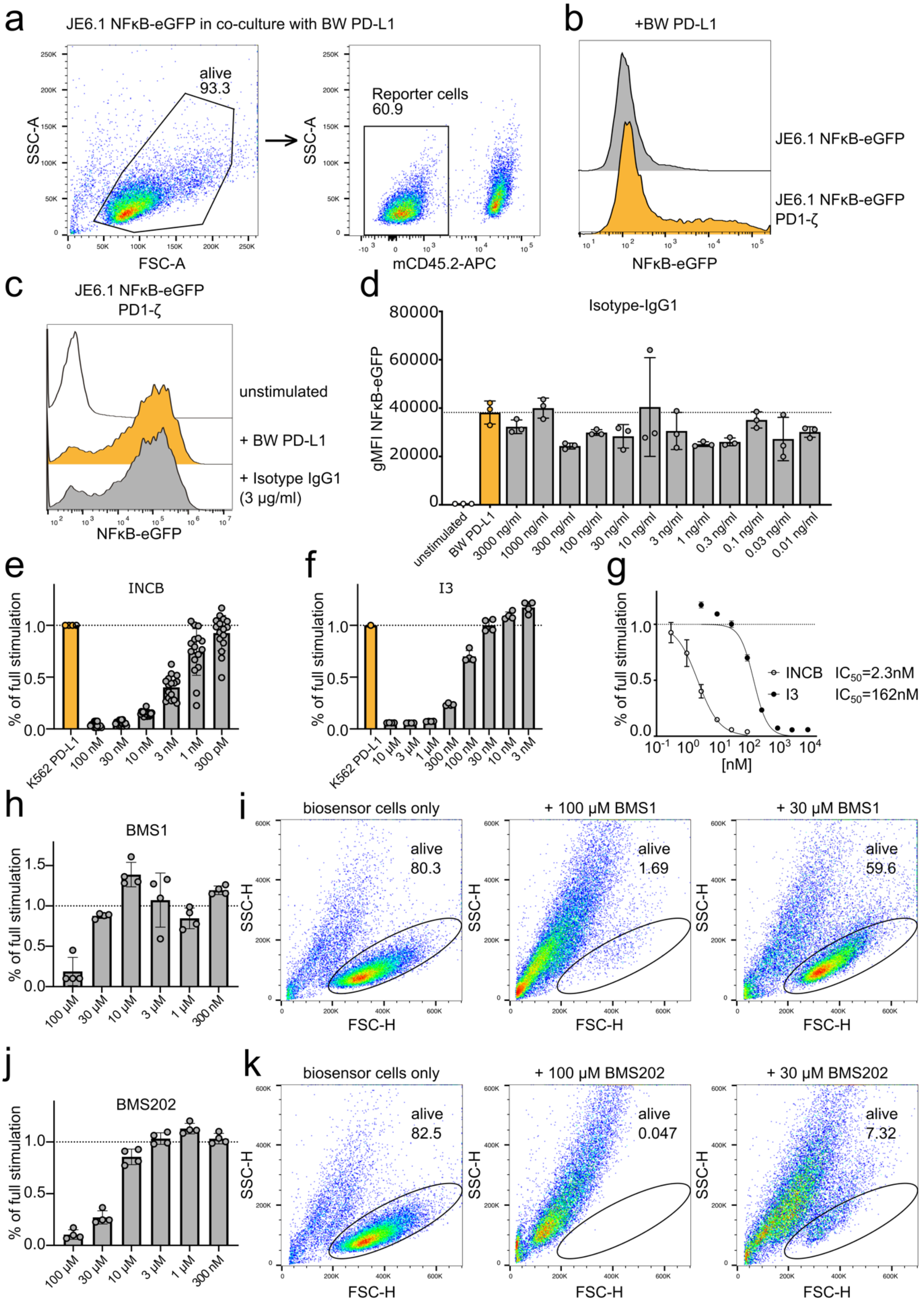

Supplementary Figure 1 ***Supplemental data of PD1-ζ biosensor cells.*** **a** Exemplary gating strategy for biosensor assays with BW stimulator cells. Viable cells were gated by FSC-A/SSC-A. Subsequently, BW-stimulator cells were stained with a APC labeled antibody to murine CD45 to allow for the identification of the APC-negative population as biosensor cells. **b** Representative histograms showing reporter gene expression of JE6.1 NFκB-eGFP and JE6.1 NFκB-eGFP PD1-ζ biosensor cells cultured with BW PD-L1 (data presented as NFκB-eGFP FI, n=2 experiments in duplicates, pooled data depicted in Fig. 1c). **c,d** Effect of isotype-control (IgG1) antibody on the stimulation of JE6.1 NFκB-eGFP PD-1-ζ biosensor cells. **c** Representative histograms JE6.1 NFκB-eGFP PD1-ζ biosensor cells under the indicated stimulation conditions (data presented as NFκB-eGFP FI). **d** Graph shows triplicate values of one experiment with mean ±SD of gMFI NFκB-eGFP. **e,f** JE6.1 NFκB-eGFP PD-1-ζ biosensor cells were stimulated by K562 PD-L1 alone or with addition of small molecule blocking compounds (**e** INCB, n=4 experiments, each 4 replicates with mean ±SD; **f** I3, n=1 experiment, 4 replicates with mean ±SD). Data was normalized to full stimulation by K562 PD-L1. Raw gMFI values are provided in the Source Data sheet. **g** Non-linear regression curve fitting with IC_{50} calculation for INCB and I3. **h** Blocking activity of BMS1 compound (n=1 experiment, 4 replicates with mean ±SD). Data was normalized to full stimulation by K562 PD-L1. Raw gMFI values are provided in the Source Data sheet. **i** Representative dot plots depicting FSC-H/SSC-H gating of JE6.1 NFκB-eGFP PD1-ζ biosensor cells alone or after incubation with BMS1. **j,k** Blocking activity of BMS202 compound and FSC-H/SSC-H gating analogous to **h** and **i**.

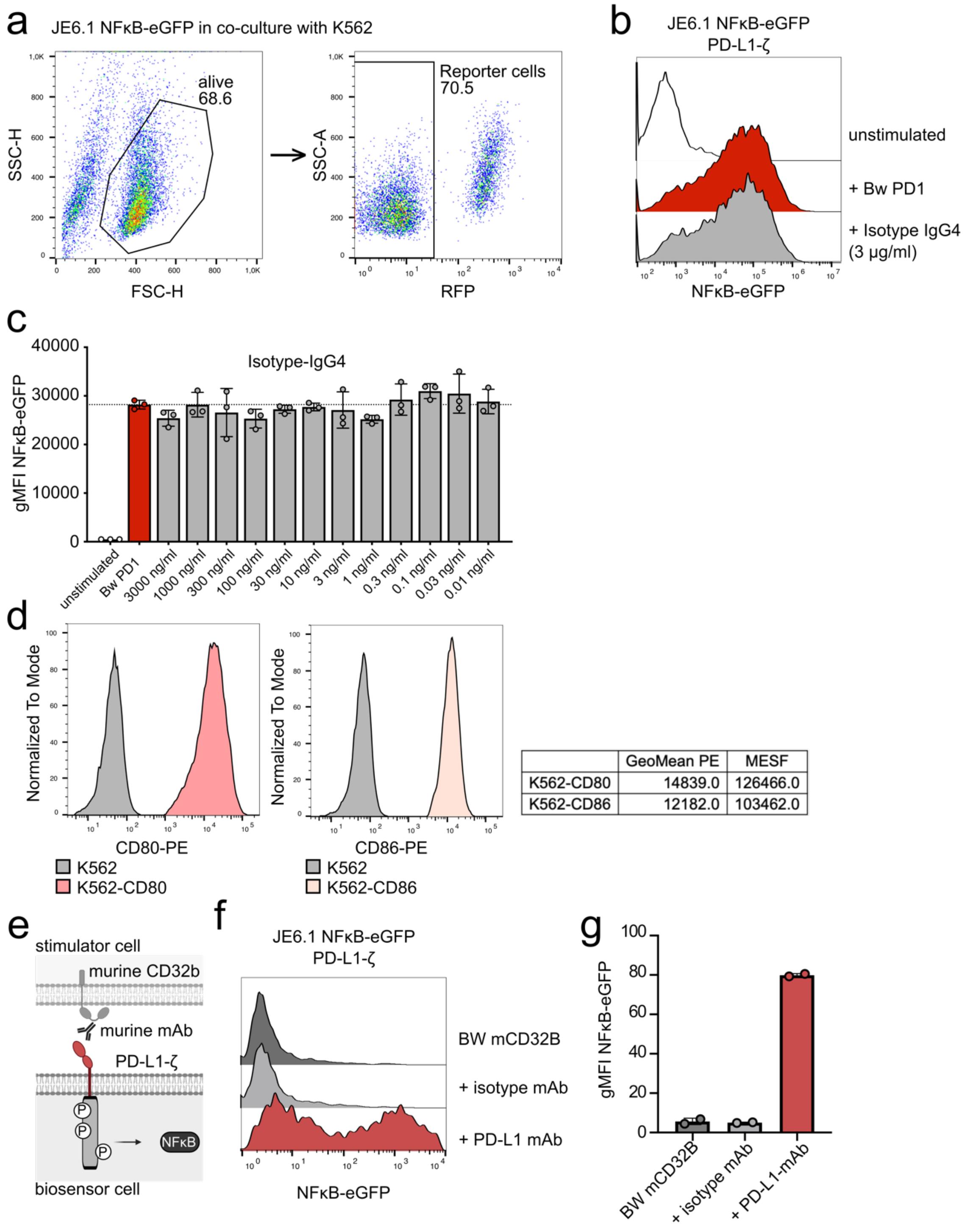

Supplementary Figure 2 ***Supplemental data of PD-L1-ζ biosensor cells.*** **a** Exemplary gating strategy for biosensor assays with RFP$^+$K562 stimulator cells. Viable cells were gated by FSC-A/SSC-A. Biosensor cells were identified as RFP-negative cells. **b,c** Effect of isotype-control (IgG4) antibody on the stimulation of JE6.1 NFκB-eGFP PD-L1-ζ biosensor cells. **b** Representative histograms showing reporter gene expression of JE6.1 NFκB-eGFP PD-L1-ζ biosensor cells under the indicated stimulation conditions (data presented as NFκB-eGFP FI). **c** Graph shows triplicate values of one experiment with mean ±SD of gMFI NFκB-eGFP. **d** Expression of CD80 (left panel) and CD86 (right panel) on K562 CD80 and K562 CD86 respectively. Molecule count was quantified using a Quantum™ MESF kit. GMFI-PE and Molecules of Equivalent Soluble Fluorochrome (MESF) values are shown in the table. **e** Scheme depicting PD-L1-ζ biosensor triggered by antibodies crosslinked via murine CD32B expressing stimulator cells. **f,g** Co-culture of JE6.1 NFκB-eGFP PD-L1-ζ biosensors with BW mCD32B cells alone or with isotype-control or PD-L1 antibodies **f** Representative histograms JE6.1 NFκB-eGFP PD-L1-ζ biosensor cells under the indicated stimulation conditions (data presented as NFκB-eGFP FI). **g** Graph shows duplicate values of one experiment with mean ±SD of gMFI NFκB-eGFP.

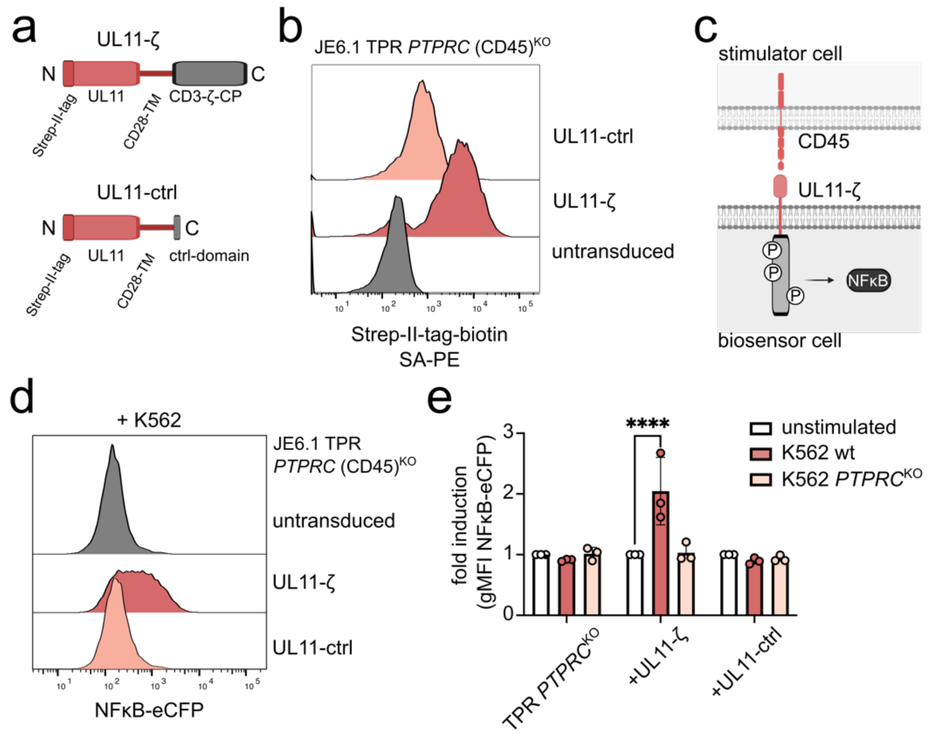

Supplementary Figure 3 ***Integration of viral cell surface molecule ectodomains into cellular biosensors.*** **a** Scheme depicting Strep-II-tagged UL11-ζ and UL11-ctrl constructs. **b** Expression of UL11-ctrl and UL11-ζ on JE6.1 $CD45^{KO}$ TPR biosensor cells. Untransduced cells serve as negative control. **c** Scheme depicting interaction on UL11-ζ on biosensor cells with CD45 on stimulator cells. **d,e** Untransduced JE6.1 TPR $CD45^{KO}$ and JE6.1 TPR $CD45^{KO}$ cells expressing UL11-ζ or UL11-ctrl were co-cultured with K562wt cells ($CD45^{+}$) or with $CD45^{KO}$-K562 cells **d** Representative histograms showing reporter gene expression of indicated biosensor/reporter cells in co-culture with K562 cells (data presented as NFκB-eGFP FI). **e** Graph shows pooled data (n=3 experiments, performed in triplicates). Triplicate means of NFκB-eCFP gMFI values were normalized to unstimulated TPR $CD45^{KO}$ cells. Data is presented as individual values, mean ±SD. Raw data is provided in the Source Data sheet. For statistical analysis two-way ANOVA with Šídák's's multiple comparisons test was performed (**** p<0.0001).

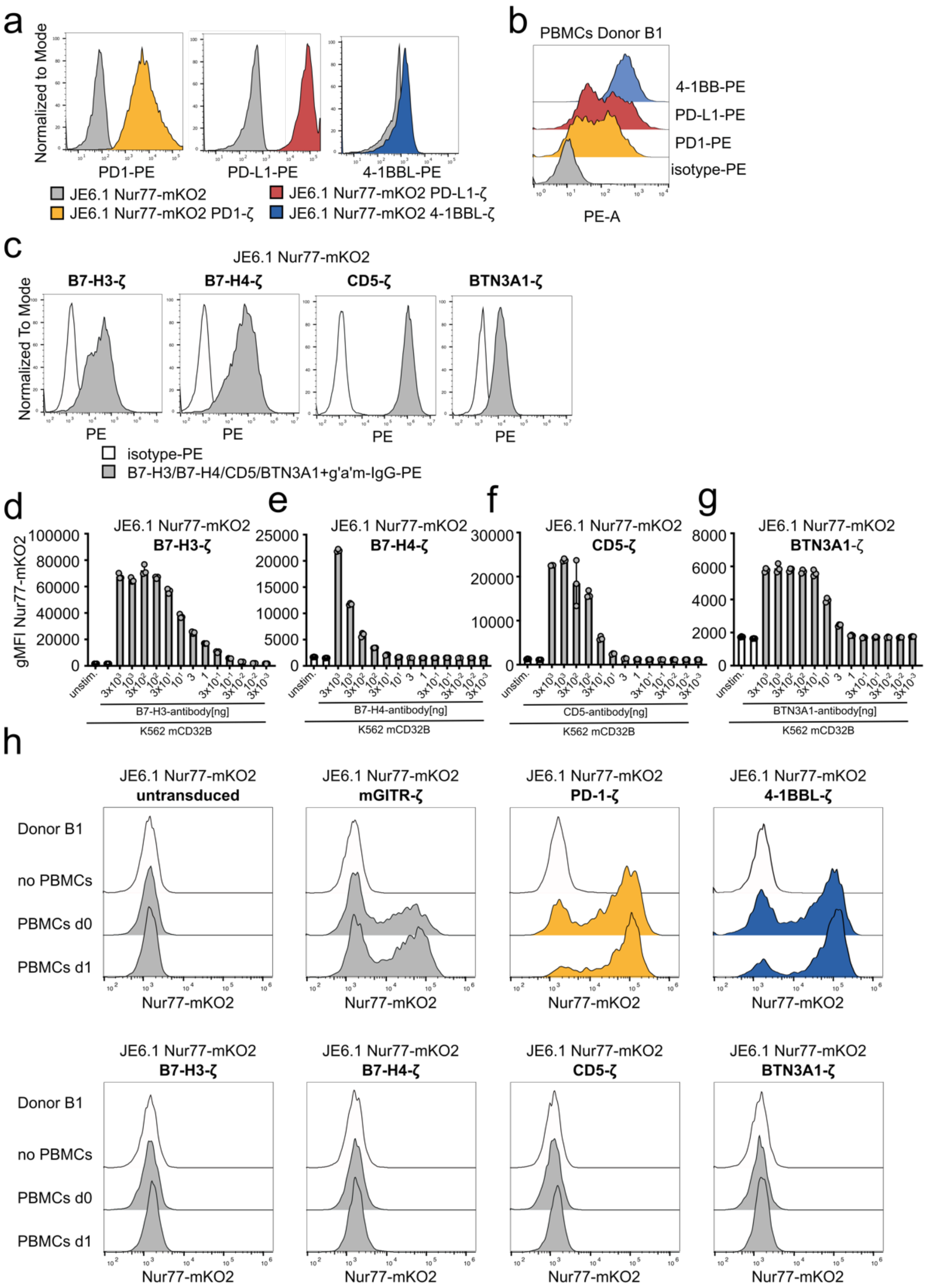

Supplementary Figure 4 ***Supplemental data of the orphan receptor screening assay.*** **a** Expression of PD-1-ζ, PD-L1-ζ and 4-1BBL-ζ on JE6.1 Nur77-mKO2 biosensor cells was verified by flow cytometry using the indicated antibodies. **b** Flow cytometry analysis of PD-1, PD-L1 and 4-1BB expression on activated human PBMCs (one representative donor is shown). **c** Expression of B7-H3-ζ, B7-H4-ζ, CD5-ζ and BTN3A1-ζ on JE6.1 Nur77-mKO.2 reporter cells. Isotype-control staining served as negative control. **d-g** JE6.1 Nur77-mKO2 biosensor cells (**d** B7-H3-ζ, **e** B7-H4-ζ, **f** CD5-ζ, **g** BTN3A1-ζ) were co-cultured with K562 expressing mCD32B alone or with the indicated amount of antibody (**d** α-B7-H3, **e** α-B7-H4, **f** α-CD5, **g** α-BTN3A1). Each assay was performed in triplicates. Data is presented as individual values with mean ±SD. **h** Representative histograms showing reporter gene expression of untransduced JE6.1 Nur77-mKO2 reporter cells and the indicated biosensor cells alone or in co-culture with resting (d0) PBMCs or activated PBMCs (d1) (data presented as Nur77-mKO2 FI) of one donor. Pooled normalized data of 3 donors is shown in Fig. 3j. The respective raw data is provided with the Souce Data file.

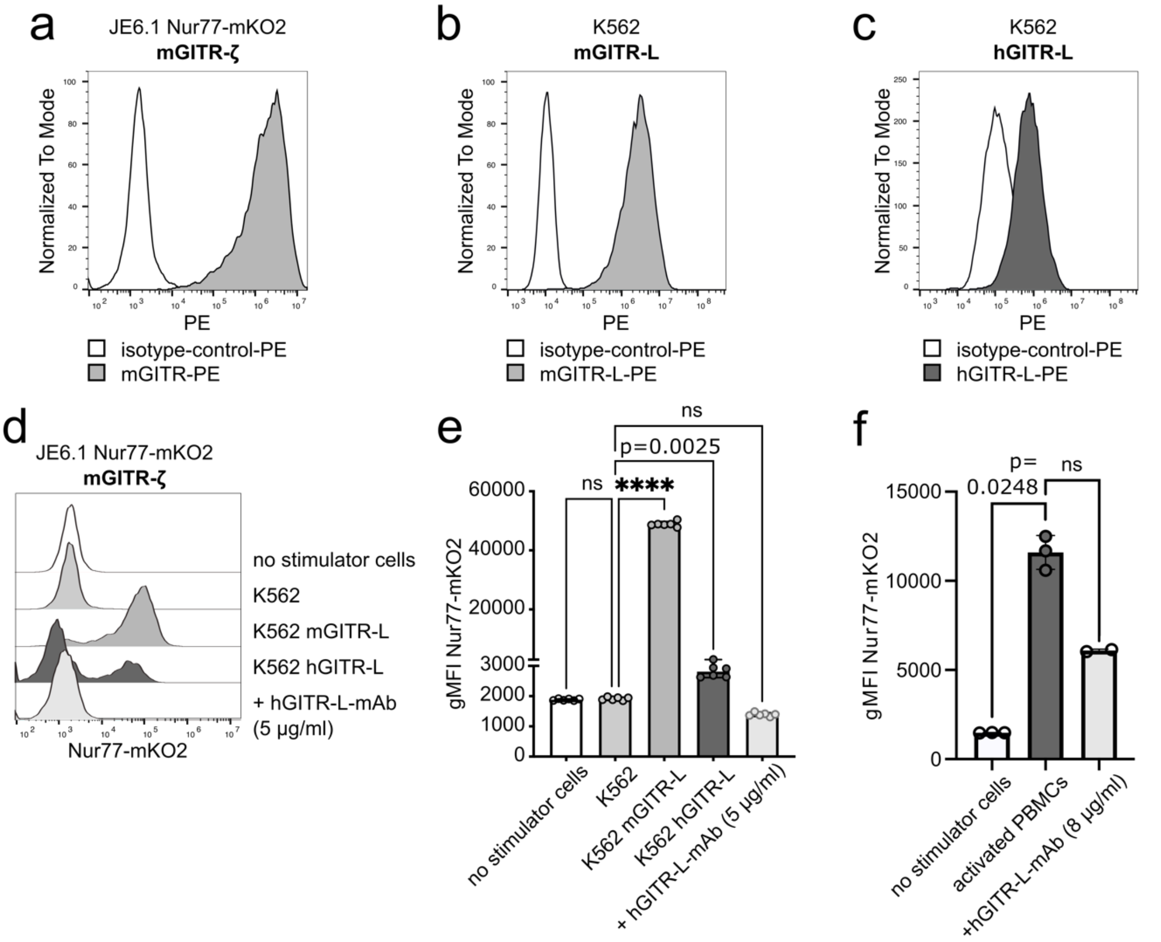

Supplementary Figure 5 ***Human GITR-L interacts with mouse (m)GITR.*** **a** Expression of mGITR-ζ, on JE6.1 Nur77-mKO2 biosensor cells. Isotype-control staining served as negative control. **b,c** Expression of mGITR-L (**b**) and hGITR-L (**c**) on K562 stimulator cells. **d,e** JE6.1 Nur77-mKO2 mGITR-ζ biosensor cells were co-cultured with the indicated stimulator cell lines. In one condition an anti-hGITR-L antibody was added. **d** Representative histograms showing reporter gene expression of JE6.1 Nur77-mKO2 mGITR-ζ biosensor cells under the indicated stimulation conditions (data presented as Nur77-mKO2 FI). **e** Graph shows replicate values of one experiment with mean ±SD of gMFI Nur77-mKO2. For statistical testing ordinary one-way ANOVA with Dunett's multiple comparisons test was used (**** $p<0.0001$; ns, not significant). **f** JE6.1 Nur77-mKO2 mGITR-ζ biosensor cells were co-cultured with anti-CD3/anti-CD28 activated PBMCs. In one condition an anti-hGITR-L antibody was added. Graph shows replicate values of one experiment (3, 2 in condition with hGITRL-mAb) with mean ±SD of gMFI Nur77-mKO2. For statistical testing Kruskal-Wallis test with Dunn's multiple comparisons test was used (ns, not significant).

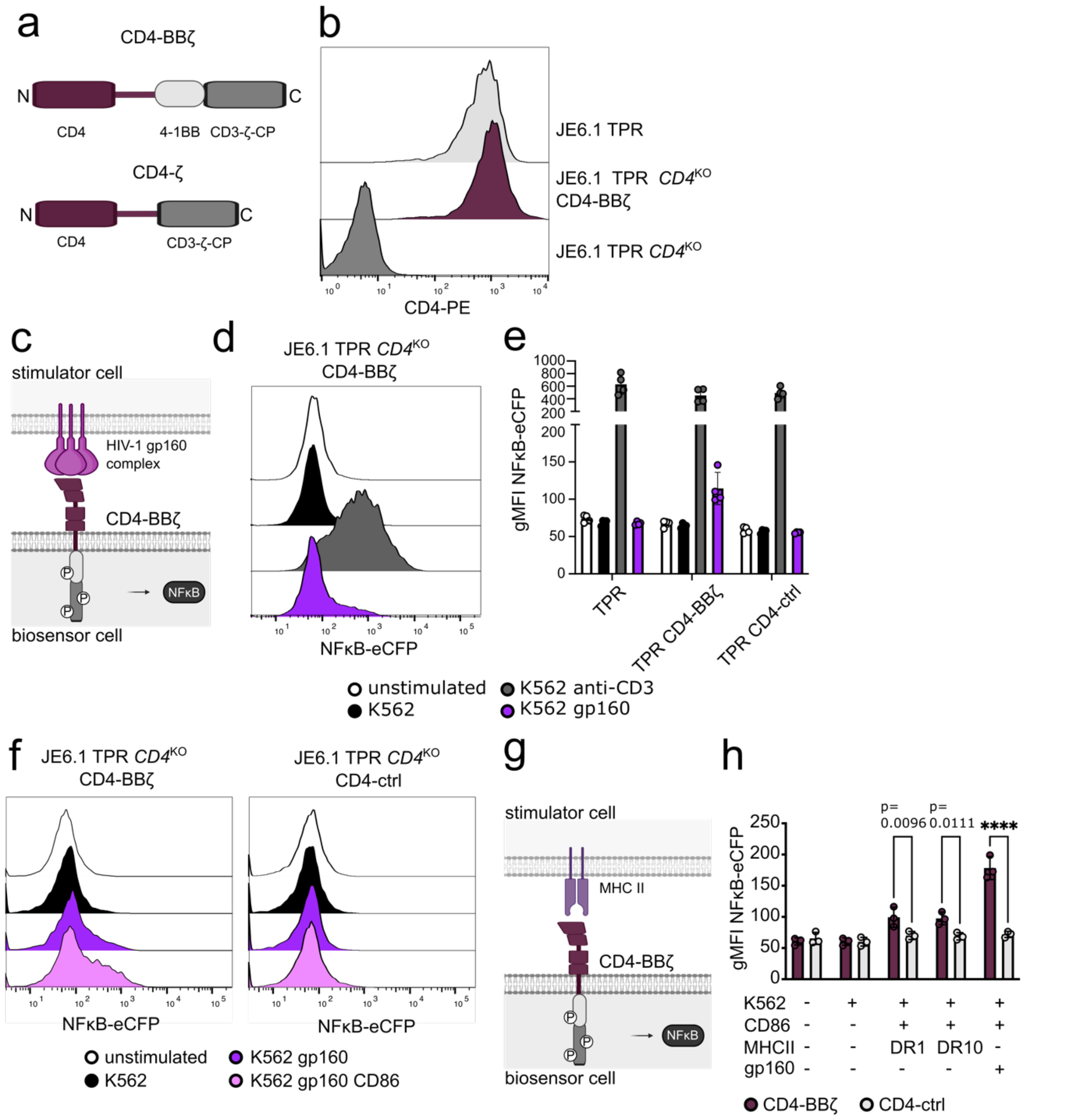

Supplementary Figure 6 ***Supplemental data of CD4-BBζ biosensor cells*** **a** Scheme depicting the CD4-BBζ and the CD4-ζ chimeric receptor. **b** Expression of CD4-BBζ on JE6.1 TPR *CD4*^{KO} biosensor cells. JE6.1 TPR and JE6.1 TPR *CD4*^{KO} served as positive and negative controls, respectively. **c** Scheme depicting interaction of gp160 complex on stimulator cell with CD4-41BBζ chimeric receptor on biosensor cells. **d,e** JE6.1 TPR, JE6.1 TPR CD4-41BBζ and JE6.1 TPR CD4-ctrl cells were co-cultured with the stimulator cells as indicated. **d** Representative histograms showing reporter gene expression of JE6.1 TPR CD4-BBζ biosensor cells after co-culture with the indicated stimulator cells (data presented as NFκB-eCFP FI). **e** Graph shows triplicate means (n=4 experiments) and overall mean ±SD of gMFI NFκB-eCFP. **f** JE6.1 TPR CD4-BBζ and JE6.1 TPR CD4-ctrl cells were cultured with the indicated stimulator cells. Representative histograms show reporter gene expression under the indicated stimulation conditions (data presented as NFκB-eCFP FI). The experiment was conducted in triplicates. **g** Scheme depicting interaction of MHC class II on stimulator cell with CD4-41BBζ chimeric receptor on biosensor cells **h** JE6.1 TPR CD4-41BBζ and JE6.1 TPR CD4-ctrl cells were co-cultured with K562 cells or K562 cells expressing the indicated molecules (n=3 experiments, performed in triplicates). For each experiment triplicate mean was calculated. Data is presented as triplicate means and overall mean ±SD of gMFI NFκB-eCFP. For statistical analysis two-way ANOVA with Šídák's's multiple comparisons test was performed (**** $p<0.0001$).

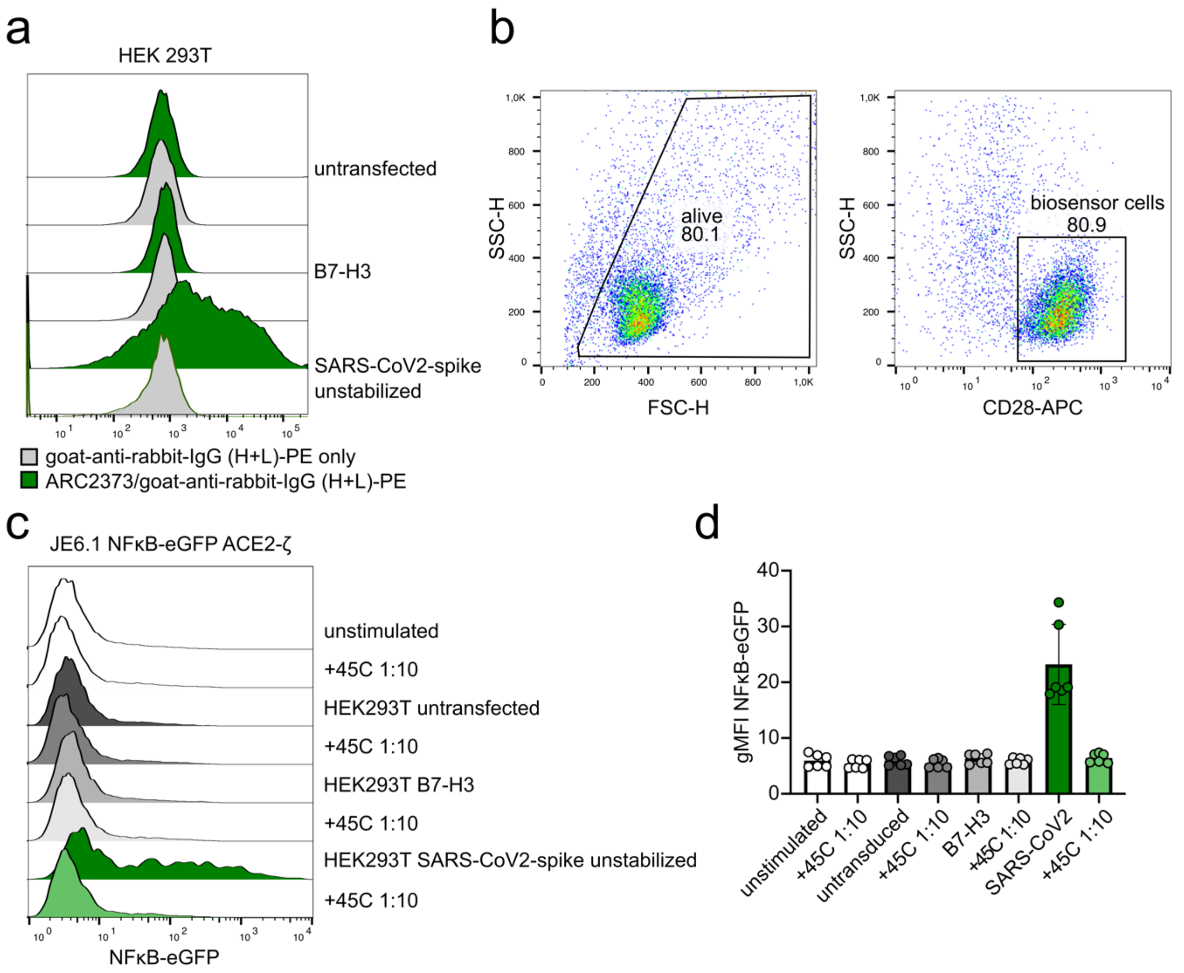

Supplementary Figure 7 ***Transient expression of SARS-CoV2 spike without stabilizing mutation induces biosensor signaling.*** **a** Expression of SARS-CoV2 spike on transiently transfected HEK293 cells. The ARC2373 monoclonal antibody in conjunction with a goat anti-rabbit secondary antibody was used for detection. Staining with secondary antibody only served as negative control. **b** Gating strategy for biosensor cell co-culture assay with HEK293. Viable cells were identified by FSC-H/SSC-H gating. Biosensor cells were identified by positivity for CD28. **c,d** JE6.1 NFκB-eGFP ACE-ζ biosensor cells were co-cultured with the indicated HEK293 cells. Each condition was also performed with serum containing neutralizing antibodies to SARS-CoV2. **c** Representative histograms show reporter gene expression under the indicated stimulation conditions (data presented as NFκB-eGFP FI). **d** Graph shows pooled data (n=2 experiments, in triplicates). Data is presented as triplicate values and mean ±SD of gMFI NFκB-eGFP.

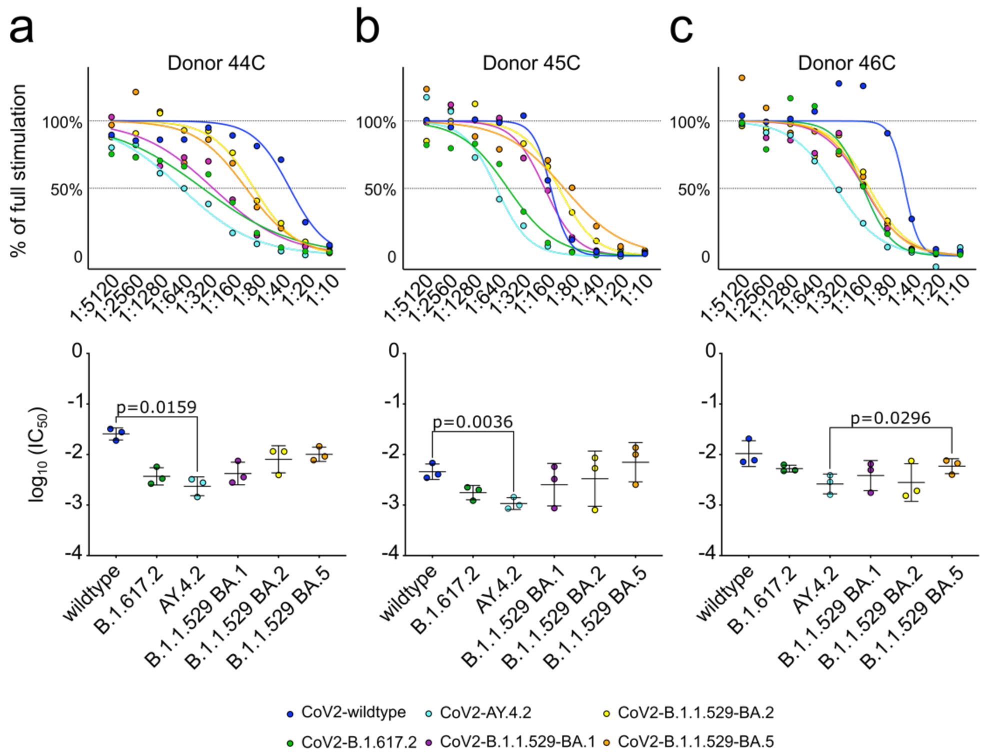

Supplementary Figure 8 ***Neutralization of SARS-CoV2 variants by donor serum.*** **a-c** Assessment of three donor sera analogous to Fig. 7d-I (**a** donor 44C, **b** donor 45C, **c** donor 46C) (n=3 experiments, performed in duplicates). Data is presented analogous to Fig. 7d-g. For statistical evaluation Shapiro-Wilk test was performed to assess normality of the data. IC_{50} values of each variant were then compared to IC_{50} values of other variants by Friedman's test with Dunn's multiple comparisons test (**a**) or repeated measure one-way ANOVA with Tukey's multiple comparison test (**b,c**).

Supplementary Tables

Primer name	Sequence	Chimeric receptor
XhoI-PD1-fw	5'-GCGCCCCTCGAGACCATGCAGATCCCACAGGCGC-3'	PD1-ζ
MluI-PD1-rv	5'-GCGCCCACGCGTCACCAGGGTTTGGAACTGGC-3'	PD1-ζ
XhoI-PDL1-fw	5'-GCGCCCCTCGAGGCCACCATGAGGATATTTGCTGTCTTTATATTC-3'	PD-L1-ζ
MluI-PDL1-rv	5'-ACGCGTACCTTTCATTTGGAGGATGTGCC-3'	PD-L1-ζ
XhoI-zeta-fw	5'-GCGCCCCTCGAGACCATGCGGGTGAAGTTCAGCAGAAG-3'	4-1BBL-ζ
4-1BBL-MluI-zeta-rv	5'-GCGCCCCTCGAGACCATGCGGGTGAAGTTCAGCAGAAG-3'	4-1BBL-ζ
zeta-MluI-4-1BBL-fw	5'-CCCCCAAGGACGCGTGAATACGCCTCTGACGCTTC-3'	4-1BBL-ζ
BamHI-4-1BBL-rv	5'-GCGCCCGGATCCTTCCGACCTCGGTGAAGG-3'	4-1BBL-ζ
XhoI-B7H3-fw	5'-GGCGGGGCTCGAGACCATGCTGCGTCGGCGGGGCAG-3'	B7-H3-ζ
MluI-B7H3-rv	5'-ACGCGTAGGCCTCTGGGGGGAATGTCA-3'	B7-H3-ζ
XhoI-B7H4-fw	5'-ACCGCCCTCGAGACCATGGCTTCCCTGGGGCAG-3'	B7-H4-ζ
MluI-B7H4-rv	5'-ACCGCCACGCGTTAGAAGCCTTTGAGTTTAGCAGC-3'	B7-H4-ζ
XhoI-CD5-fw	5'-ACCGCCCTCGAGACCATGCCCATGGGGTCTCTGC-3'	CD5-ζ
MluI- CD5-rv	5'-ACCGCCACGCGTTGGGGTTTGGATCCTGGCAT-3'	CD5-ζ
XhoI-CD46-fw	5'-GCGCCCCTCGAGACCATGGAGCCTCCCGGCCGC-3'	CD46-ζ
MluI- CD46-rv	5'-GCGCCCACGCGTCATCCAAACTGTCAAGTATTCCTTCC-3'	CD46-ζ
XhoI-hemagglutinin-fw	5'-GCGCCCCTCGAGACCATGGGCAGCCGGATCGTG-3'	measles-hemagglutinin (HG)
BamHI-hemagglutinin-V5-rv	5'-GCGCCCGGATCCGGTTGAATCCAGCCCCAGCAGAGGGTT-AGGAATAGGTTTCCCTCTGCGATTGGTTCCATCTTCC-3'	measles-HG
ΔCD150-1-fw	5'-GCAGCAACTTCCGCAGTTGAACATGCTGTGGTTTATTAC-3'	measles-HG- ΔCD150
ΔCD150-1-rv	5'-TCAACTGCGGAAGTTGCTGCGGTTGCCAAAACATATTGGAGAT-3'	measles-HG ΔCD150
ΔCD150-2-fw	5'-GCACCTTTTAGGTTGCCTATAAAGG-3'	measles-HG ΔCD150
ΔCD150-2-rv	5'-ATAGGCAACCTAAAAGGTGCAAAGTAAGAAAATGAGCGGCTTG-3'	measles-HG ΔCD150
ΔCD46-1-fw	5'-CAACAATTCATATTGGCTGACTATCCCGCC-3'	measles-HG ΔCD46
ΔCD46-1-rv	5'-TCAGCCAATATGAATTGTTGTGGTTGGATTTGTATAGG-3'	measles-HG ΔCD46
ΔCD46-2-fw	5'-ATGTTTTGTCAACCTACGATACTTCCAGGGTT-3'	measles-HG ΔCD46
ΔCD46-2-rv	5'-ATCGTAGGTTGACAAAACATATTGGAGATCTTGACC-3'	measles-HG ΔCD46
XhoI-CD4-fw	5'-GCGCCCCTCGAGACCATGAACCGGGGAGTCCCTT-3'	CD4-ζ/CD4-BBζ
MluI- CD4-rv	5'-GCGCCCACGCGTCTGGCTGCACCGGGGTGG-3'	CD4-ζ/CD4-BBζ
XhoI-V5-tag-ACE2-fw	5'-GCGCCCCTCGAGACCATGTCCTCATC-3'	ACE2-ζ
Mlul-V5-tag-ACE2-rv	5'-GCGCCCACGCGTGACTCACTGGTGG5-3'	ACE2-ζ

Supplementary Table 1 *Primers used for cloning chimeric molecules with receptor/ligand-ectodomains.*

Name	Amino acid sequence
Murine CD32B (mCD32B)	UniProtKB P08101
PD-1	UniProtKB Q15116-1 (S38F sequence conflict)
PD-L1	UniProtKB Q9NZQ7
CD80	UniProtKB P33681-1
CD86	UniProtKB P42081, V185I
PD-L1-ζ	UniProtKB Q9NZQ7-1 (aa 1-238)-NA-CD28 TM-CD3ζ
PD-1-ζ	UniProtKB Q15116-1 (aa 1-170, S38F sequence conflict)-NA- CD28 TM-CD3ζ
4-1BB	UniProtKB Q07011
4-1BBL	UniProtKB P41273-1
4-1BBL-ζ	M-CD3ζ-TR- UniProtKB P41273-1 (aa 2-254)
UL11-ζ	UniProtKB Q6SWB9-1 (aa 1-31)-GSNWSHPQFEK (Strep-II-tag)- UniProtKB Q6SWB9-1 (aa 32-224)-NA-CD28 TM- CD3ζ
UL11-ctrl	UniProtKB Q6SWB9-1 (aa 1-31)-GSNWSHPQFEK (Strep-II-tag)- UniProtKB Q6SWB9-1 (aa 32-224)-NA-CD28 TM- UniProtKB Q15116-1 (aa 192-208)
murine GITR-ζ (mGITR-ζ)	UniProtKB O35714 (aa1-153)-NA-CD28 TM-CD3ζ
murine GITR-L (mGITR-L)	UniProtKB Q7TS55
human GITR-L (hGITR-L)	UniProtKB Q9UNG2
B7-H3-ζ	UniProtKB Q5ZPR3-1 (aa1-168, aa387-466)-NA-CD28 TM-CD3ζ (2Ig-domains)
B7-H4-ζ	UniProtKB Q7Z7D3 (aa 1-259)-NA-CD28 TM-CD3ζ
CD5-ζ	UniProtKB P06127 (aa 1-372, P224L natural variant)-NA-CD28 TM-CD3ζ
BTN3A1-ζ	UniProtKB O00481 (aa1-255)-CD3ζ(aa31-164)
Measles hemagglutinin	Addgene plasmid #83817 (pCG-HcΔ18, gift from Jakob Reiser, aa 1-599)-GKPIPNPLLGLDST (V5-tag)[23]
Measles hemagglutinin ΔCD46	Measles hemagglutinin, V433S, A509S
Measles hemagglutinin ΔCD150	Measles hemagglutinin, Y511A, D512A, H515A, Y535A
CD46-ζ	UniProtKB P15529-12 (aa 1-313)-DA-CD28 TM -CD3ζ
CD46-ctrl	UniProtKB P15529-12 (aa 1-313)-DA-CD28 TM- UniProtKB Q15116-1 (aa 192-208)
BaL-HIV-gp160	Addgene plasmid #100919 (pCEP-BaLgp160, gift from Erik Procko, aa 1-850)[24]
CD4-41BBζ	UniProtKB P01730-1 (aa 1-396)-DA-CD28 TM- 4-1BB-CD3ζ
CD4-ζ	UniProtKB P01730-1 (aa 1-396)-DA-CD28 TM-CD3ζ
CD4-ctrl	UniProtKB P01730-1 (aa 1-396)-DA-CD28 TM- UniProtKB Q15116-1 (aa 192-208)
HLA DR1	HLA-DRA1*01/B1*01:01:01
HLA DR10	HLA-DRA1*01/B1*10:01:01:01
ACE2-ζ	UniProtKB Q9BYF1-1 (aa 1-17)-2xEQKLISEEDL(c-myc-tag)- UniProtKB Q9BYF1-1 (aa 18-740)-HA- CD28 TM- CD3ζ
SARS-CoV-2-spike-wild type	UniProtKB P0DTC2-1 (aa 1-12)- GSNWSHPQFEK (Strep-II-tag)- UniProtKB P0DTC2-1 (aa 13-1273)- del682-683, del685, K986P, V987P
SARS-CoV-2-spike-B.1.617.2	SARS-CoV-2-spike-wildtype, T19R, G142D, E156G, Δ157-158, L425R, T478K, D614G, P681R, D950N (R685S)
SARS-CoV-2-spike-AY.4.2	SARS-CoV-2-spike-B.1.617.2, T91I, Y145H, A222V
SARS-CoV-2-spike-B.1.1.529 BA.1	SARS-CoV-2-spike-wildtype, A67V, Δ69-79, T95I, G142D, Δ143-145, N211I, Δ212 215EPEins, G339D, S371L, S373P, S375F, K417N, N440K, G446S, S477N, T478K, E484A, Q493R, G496S, Q498R, N501Y, Y505H, T547K, D614G, H655Y, N679K, P681H, N764K, D796Y, N856K, Q954H, N969K, L981F
SARS-CoV-2-spike-B.1.1.529 BA.2	SARS-CoV-2-spike-wildtype, T19I, L24S, 25PPAins, G142D, V213G, G339D, S371L, S373P, S375F, T376A, D405N, R408S, K417N, N440K, S477N, T478K, E484A, Q493R, Q498R, N501Y, Y505H, D614G, H655Y, N679K, P681H, N764K, D796Y, Q954H, N969K
SARS-CoV-2-spike-B.1.1.529 BA.5	SARS-CoV-2-spike-wildtype, T19I, L24S, Δ25-27, Δ69-70, G142D, V213G, G339D, S371F, S373P, S375F, T376A, D405N, R408S, K417N, N440K, G446S, L452R, S477N, T478K, E484A, F486V, Q498R, N501Y, Y505H, T547K, D614G, H655Y, N679K, P681H, N764K, D796Y, Q954H, N969K

Supplementary table 2 *Amino acid sequences of unmodified, modified and chimeric molecules expressed on stimulator or reporter cells.*

Antibody/Protein	Clone	Lot/Batch number	Catalog number	Supplier	Dilution (final)
PE-Isotype	MOPC-21	B289689	400114	Biolegend, San Diego, CA	1:200
APC mouse-CD45.2	104	B338570	109814	Biolegend	1:400
APC-CD46	TRA-2-10	B329350	352401	Biolegend	1:50
APC-CD28	CD28.2	B218747	302912	Biolegend	1:50
Biotin-Strep-II-tag	5A9F9	2107K043	A01737-100	GenScript, Piscataway NJ	1:100
CD4-Fc Tag		179-9BEF1-Q8	CD4-H5259	Acro Biosystems, Newark, DE	conc. indicated
FITC-c-myc	SH1-26E7.1.3	5200905664	130-116-485	Miltenyi Biotec, Bergisch Gladbach, GER	1:16
PE-4-1BB	4B4-1	B266071	309803	Biolegend	1:50
PE-4-1BB-L	5F4	B334584	311504	Biolegend	1:50
PE-CD4, unconjugated-CD4	OKT4	B304227	317410	Biolegend	1:50/conc. indicated
PE-CD45	2D1	B311739	368510	Biolegend	1:50
PE-F(ab')2 goat anti mouse IgG (H+L)	Polyclonal	152883	115-116-146	Jackson ImmunoResearch, West Grove, PA	1:100
PE-PD-L1, unconjugated-PD-L1	29E.2A3	B236176	329706	Biolegend	1:50/conc. indicated
PE-PD-1	EH12.2H7	B329771	329906	Biolegend	1:50
PE-Streptavidin		9123795	554061	BD Biosciences, Franklin Lakes, NJ	1:50
PE-V5 Tag	TCM5	2622508	12-6796-42	Invitrogen, Waltham, MA	1:50
PE-CD80	2D10	B301546	305208	Biolegend	1:50
PE-CD86	IT 2.2	B356097	305405	Biolegend	1:50
B7H3	7517			Provided by Otto Majdic	conc. indicated
B7H4	973816/MAB66761	CKYL012306A	MAB65761	R&D Systems, Minneapolis, MN	conc. indicated
CD5	L17F12	B342830	364002	Biolegend	conc. indicated
hGITRL	109101/MAB6941	DTF052305A	MAB6941	R&D Systems	conc. indicated
PE-hGITRL	REA841	5210205310	130-113-038	Miltenyi Biotec	conc. indicated
PE-mGITRL	MIH44	0238572	563541	BD Biosciences	conc. indicated
PE-mGITR	DTA-1	B288817	126309	Biolegend	conc. indicated
hBTN3A1/2/3	849203/MAB7136	CIYN0122031	MAB7136	R&D Systems	conc. indicated
SARS-CoV-2 Spike	ARC2373	VJ3085877	MA5-36087	Thermo Fisher Scientific	1:50
PE-F(ab')2 goat anti rabbit IgG (H+L)	polyclonal	160005	111-116-144	Jackson ImmunoResearch	1:100

Supplementary table 3 *Conjugated and unconjugated antibodies and other reagents used for staining, flow cytometry*

Name	Product-type	Supplier/Contributor	Lot/Batch/Charge number	Catalog number
Privigen	Intravenous immunoglobulin	CSL Behring, King of Prussia, PA	P100426756	43239
octagam	Intravenous immunoglobulin	Octapharma, Lachen, CH	K218A8542	2-00346
SIM.2	Anti-human CD4 mAb	NIH HRP, Dr. James E.K. Hildreth	170136	ARP-723
NIH45-46 G54W	Anti-HIV-1 gp120 mAb	NIH HRP, Dr. Pamela Bjorkman[32]	160101	ARP-12174
VRC-CH31	Anti-HIV-1 Envelope CD4 Binding Site mAb	NIH HRP, Drs. Barton F. Haynes and Hua-Xin Liao	140161	ARP-12565
Regdanvimab	Therapeutic anti-SARS-CoV-2-spike mAb	Celltrion, Incheon, RK	21006342	
Tixagevimab (AZD8895)	Prophylactic anti-SARS-CoV-2-spike mAb	AstraZeneca, Cambridge, UK	CAAK	
Cilgavimab (AZD1061)	Prophylactic anti-SARS-CoV-2-spike mAb	AstraZeneca	CAAK	
Avelumab (Bavencio)	Therapeutic PD-L1 mAb	Merck, Darmstadt, GER	AU026181	
Atezolizumab (Tecentriq)	Therapeutic PD-L1 mAb	Roche, Basel, CH	H0105	
Nivolumab (Opdivo)	Therapeutic PD-1 mAb	Bristol-Myers Squibb GmbH & Co	AAT5725	
Ultra LEAF IgG1 Isotype (clone QA16A12)	Isotype-control antibody	Biolegend	B372653	403502
Ultra LEAF IgG4 Isotype (clone QA16A15)	Isotype-control antibody	Biolegend	B305479	403702
Utomilumab (PF-05082566)	Therapeutic 4-1BB agonistic mAb	Creative Biolabs, Shirley, NY	CB1900108	TAB-457CQ
Urelumab (BMS-663513)	Therapeutic 4-1BB agonistic mAb	Creative Biolabs	CB0421JF01	TAB179
Human 4-1BB/TNFRSF9/CD137	Polyclonal antibodies	Biotechne (R&D Systems), Minneapolis, MN	CCO0217041	AF838
INCB086550 (INCB)	Small molecule inhibitor	MedChemExpress, Monmouth Junction, NJ	226429	HY-134884
PD-1/PD-L1 Inhibitor 3 (I3)	Small molecule inhibitor	Selleckchem, Houston, TX	S815803	S8158
BMS1	Small molecule inhibitor	Selleckchem	S791101	S7911
BMS202	Small molecule inhibitor	Selleckchem	S791202	S7912
ORTHOCLONE OKT 3 1mg/ml	Monoclonal antibody (PBMC activation)	JANNSEN-CILAG, Johnson & Johnson, New Brunswick, NJ	aAHSTU00	
Ultra-LEAF Purified anti-human CD28 Antibody (clone CD28.2)	Monoclonal antibody (PBMC activation)	Biolegend	B313286	302933

Supplementary table 4 *Reagents used in receptor/ligand interaction assays*
