# Supplementary material for: Interrogating ligand-receptor interactions using highly sensitive cellular biosensors
Source: Nat Commun. 2023 Nov 28;14:7804. doi: 10.1038/s41467-023-43589-1 (PMC10684770; doi:10.1038/s41467-023-43589-1)
Supplement: Supplementary file 3 — Reporting Summary [file 41467_2023_43589_MOESM3_ESM.pdf]

nature portfolio

Corresponding author(s): Judith Leitner
Peter Steinberger

Last updated by author(s): 29.10.2023

Reporting Summary

Nature Portfolio wishes to improve the reproducibility of the work that we publish. This form provides structure for consistency and transparency in reporting. For further information on Nature Portfolio policies, see our Editorial Policies and the Editorial Policy Checklist.

Statistics

For all statistical analyses, confirm that the following items are present in the figure legend, table legend, main text, or Methods section.

n/a	Confirmed	
☐	☒	The exact sample size (*n*) for each experimental group/condition, given as a discrete number and unit of measurement
☐	☒	A statement on whether measurements were taken from distinct samples or whether the same sample was measured repeatedly
☐	☒	The statistical test(s) used AND whether they are one- or two-sided<br>*Only common tests should be described solely by name; describe more complex techniques in the Methods section.*
☐	☒	A description of all covariates tested
☐	☒	A description of any assumptions or corrections, such as tests of normality and adjustment for multiple comparisons
☐	☒	A full description of the statistical parameters including central tendency (e.g. means) or other basic estimates (e.g. regression coefficient) AND variation (e.g. standard deviation) or associated estimates of uncertainty (e.g. confidence intervals)
☐	☒	For null hypothesis testing, the test statistic (e.g. *F*, *t*, *r*) with confidence intervals, effect sizes, degrees of freedom and *P* value noted<br>*Give P values as exact values whenever suitable.*
☒	☐	For Bayesian analysis, information on the choice of priors and Markov chain Monte Carlo settings
☒	☐	For hierarchical and complex designs, identification of the appropriate level for tests and full reporting of outcomes
☐	☒	Estimates of effect sizes (e.g. Cohen's *d*, Pearson's *r*), indicating how they were calculated

Our web collection on statistics for biologists contains articles on many of the points above.

Software and code

Policy information about availability of computer code

Data collection	CellQuest V6.0 (BDBioscience,NJ,USA), FACSDiva V9.0 (BDBioscience,NJ,USA), CytExpert V2.4 (Beckman Coulter, CA USA)
Data analysis	FlowJo V10.7.1 (BD Bioscience, NJ, USA), Prism V9 (GraphPad, MA, USA), TIDE online tool (http://shinyapps.datacurators.nlitide/)

For manuscripts utilizing custom algorithms or software that are central to the research but not yet described in published literature, software must be made available to editors and reviewers. We strongly encourage code deposition in a community repository (e.g. GitHub). See the Nature Portfolio guidelines for submitting code & software for further information.

Data

Policy information about availability of data

All manuscripts must include a data availability statement. This statement should provide the following information, where applicable:

- Accession codes, unique identifiers, or web links for publicly available datasets
- A description of any restrictions on data availability
- For clinical datasets or third party data, please ensure that the statement adheres to our policy

A Source Data file containing all raw geometric mean fluorescence intensity (gMFI) values, normalization steps and statistical tests is provided as supplementary file with this paper. Supplementary Table 2 lists the UniProtKB entries of all proteins expressed in cell lines for this study. All amino acid modifications are also listed in Supplementary Table 2. All UniProtKB entries can be acccessed on the UniProt online database (https://www.uniprot.org).

Research involving human participants, their data, or biological material

Policy information about studies with human participants or human data. See also policy information about sex, gender (identity/presentation), and sexual orientation and race, ethnicity and racism.

Reporting on sex and gender	Information of study participant sex was obtained by self-reporting at the time of sample collection after obtaining written consent(Sera:164w,46m,1d;PBMC:1m,2unknown).However,since there was no intra-or intercohort comparisons and no aspects known to be influenced by sex were investigated, the information on sex was not deemed relevant and is not reported with this study. Data on gender was not collected.
Reporting on race, ethnicity, or other socially relevant groupings	No socially constructed or socially relevant categorizations were performed for this study.
Population characteristics	The cohort of serum donors was recruited at the FH Campus Vienna (n=193) and the Institute of Immunology of the Medical University of Vienna (n=18)(n total =211). Donors at the FH Campus Vienna were analyzed for the presence of SARS-CoV2 specific antibodies using the electrochemiluminescence immunoassay (n=193). Serum of 181 of the 193 FH Campus donors was available for further analysis with the biosensor assay. Age of serum donors was available for 193 of 211 donors (median 22a, range 18-61a). Age of PBMC donors was not reported. Furthermore for some patients self reported "antigen exposure" (contact, vaccination, self reported testing) prior to sampling was assessed. Of all serum donors 63 had a reported contact to a SARS-CoV2 positive person prior to sampling. 126 had no contact, data of 22 donors was unavailable. 73 donors had a reported vaccination against SARS-CoV2 prior to sampling, 134 were unvaccinated, 3 did not report vaccination status. 32 donors reported having tested positve for SARS-CoV2 (testing methodology not specified) prior to sampling. Most donors were sampled once, 9 donors were sampled repeatedly (2-7 times), however not systematically.<br>For figure 4g, measles immunization status was obtained from one donor. For figure 6g, SARS-CoV2 immunization status was obtained from one donor at 3 time points. The serum samples analysed in Fig. 6h/i were tested to compare the results from the biosensor assay to established assays and therefore any covariate would have influenced both assays simultaneously. No covariates were considered for serum samples in Supplementary Figure 8. Therefore there is no statement on how interpersonal antibody titers may have come about.
Recruitment	Serum donors were acquired by public announcements, PBMC donors were recruited through the Vienna Red Cross blood donor service. Written informed consent was obtained from all donors.
Ethics oversight	The study with human sera was approved by the ethics committee of the Medical University of Vienna under the registration number 2262/2020. Human PBMCs were obtained under approval by the ethics committee of the Medical University of Vienna under the registration number 1183/2016.

Note that full information on the approval of the study protocol must also be provided in the manuscript.

Field-specific reporting

Please select the one below that is the best fit for your research. If you are not sure, read the appropriate sections before making your selection.

☒ Life sciences ☐ Behavioural & social sciences ☐ Ecological, evolutionary & environmental sciences

For a reference copy of the document with all sections, see nature.com/documents/nr-reporting-summary-flat.pdf

Life sciences study design

All studies must disclose on these points even when the disclosure is negative.

Sample size	There was no sample size calculation specifically for this study. Human sera collected for a separate study and all available samples were reanalyzed with the biosensor assay. For the neutralization test the maximal number of samples by available resources for research purposes was analyzed by the Department of Virology of the Medical University of Vienna.
Data exclusions	Of 78 serum donors with reactivity in the Elecsys® Anti-SARS-CoV-2 electrochemiluminescence immunoassay one did not show neutralization in the biosensor reporter asssay. This sample was therefore excluded for the correlation as it was not plottable. For a repeat of Fig. 5g (not shown, data in the Source Data file) some replicates strongly diverged from the other replicates, most likely due to a measuring error.
Replication	Replications were performed as indicated in the manuscript. All replicates conducted are presented either in the figures or the Source Data file. There were no unsuccessful replication attempts.
Randomization	There was no allocation to different experimental groups and therefore no randomization was performed.
Blinding	Serum samples were first analyzed by the electro chemiluminescence assay, followed by the biosensor assay and lastly the neutralization test. Results from each test were made available to the performing researchers only after analysis. Blinding was not performed because assays were set up and measured by the same researcher.

Reporting for specific materials, systems and methods

We require information from authors about some types of materials, experimental systems and methods used in many studies. Here, indicate whether each material, system or method listed is relevant to your study. If you are not sure if a list item applies to your research, read the appropriate section before selecting a response.

Materials & experimental systems

n/a	Involved in the study
☐	☒ Antibodies
☐	☒ Eukaryotic cell lines
☒	☐ Palaeontology and archaeology
☒	☐ Animals and other organisms
☒	☐ Clinical data
☒	☐ Dual use research of concern
☒	☐ Plants

Methods

n/a	Involved in the study
☒	☐ ChIP-seq
☐	☒ Flow cytometry
☒	☐ MRI-based neuroimaging

Antibodies

Antibodies used

PE-Isotype, clone MOPC-21, Lot/Batch number: B289689, Cat.-No.: 400114 Biolegend, San Diego, CA
APC mouse-CD45.2, clone 104, Lot/Batch number: B338570, Cat.-No.: 109814 Biolegend
APC-CD46, clone TRA-2-10, Lot/Batch number: B329350, Cat.-No.: 352401 Biolegend
APC-CD28, clone CD28.2, Lot/Batch number: B218747, Cat.-No.: 302911 Biolegend
Biotin-Strep-II-tag, clone 5A9F9, Lot/Batch number: 2107K043, Cat.-No.: A01737-100 GenScript, Piscataway NJ
FITC-c-myc, clone SH1-26E7.1.3, Lot/Batch number: 5200905664, Cat.-No.: 130-116-485 Miltenyi Biotec, Bergisch Gladbach, GER
PE-4-1BB, clone 4B4-1, Lot/Batch number: B266071, Cat.-No.: 309803 Biolegend
PE-4-1BB-L, clone 5F4, Lot/Batch number: B334584, Cat.-No.: 311504 Biolegend
PE-CD4, unconjugated-CD4, clone OKT4, Lot/Batch number: B304227, Cat.-No.: 317410 Biolegend
PE-CD45, clone 2D1, Lot/Batch number: B311739, Cat.-No.: 368510 Biolegend
PE-F(ab')2 goat anti mouse IgG (H+L), polyclonal, Lot/Batch number: 152883, Cat.-No.: 115-116-146 Jackson ImmunoResearch, West Grove, PA
PE-PD-L1, unconjugated-PD-L1, clone 29E.2A3, Lot/Batch number: B236176, Cat.-No.: 329706 Biolegend
PE-PD-1, clone EH12.2H7, Lot/Batch number: B329771, Cat.-No.: 329906 Biolegend
PE-V5 Tag, clone TCM5, Lot/Batch number: 2622508, Cat.-No.: 12-6796-42 Invitrogen, Waltham, MA
PE-CD80, clone 2D10, Lot/Batch number: B301546, Cat.-No.: 305208 Biolegend
PE-CD86, clone IT 2.2, Lot/Batch number: B356097, Cat.-No.: 305405 Biolegend
B7H3, clone 7517 Provided by Otto Majdic
B7H4, clone 973816, Lot/Batch number: CKYL012306A, Cat.-No.: MAB65761 R&D Systems, Minneapolis, MN
CD5, clone L17F12, Lot/Batch number: B342830, Cat.-No.: 364002 Biolegend
hGITRL, clone 109101, Lot/Batch number: DTF052305A, Cat.-No.: MAB6941 R&D Systems
PE-hGITRL, clone REA841, Lot/Batch number: 5210205310, Cat.-No.: 130-113-038 Miltenyi Biotec
PE-mGITRL, clone MIH44, Lot/Batch number: 0238572, Cat.-No.: 563541 BD Biosciences
PE-mGITR, clone DTA-1, Lot/Batch number: B288817, Cat.-No.: 126309 Biolegend
hBTN3A1/2/3, clone 849203, Lot/Batch number: CIYN0122031, Cat.-No.: MAB7136 R&D Systems
SARS-CoV-2 Spike, clone ARC2373, Lot/Batch number: VJ3085877, Cat.-No.: MA5-36087 Thermo Fisher Scientific
PE-F(ab')2 goat anti rabbit IgG (H+L), polyclonal, Lot/Batch number: 160005, Cat.-No.: 111-116-144 Jackson ImmunoResearch
Ultra LEAF IgG1 Isotype (clone QA16A12) Isotype-control antibody, Biolegend, Lot/Batch number: B372653, Cat.-No.: 403502
Ultra LEAF IgG4 Isotype (clone QA16A15) Isotype-control antibody, Biolegend, Lot/Batch number: B305479, Cat.-No.: 403702
ORTHOCLONE OKT3, monoclonal antibody (PBMC activation) JANNSEN-CILAG, Johnson & Johnson, New Brunswick, NJ, Lot/Batch number: aAHSTU00
Ultra-LEAF Purified anti-human CD28 Antibody (clone CD28.2), monoclonal antibody (PBMC activation), Biolegend, Lot/Batch number: B313286, Cat.-No.: 302933
SIM.2 Anti-human CD4 mAb, provided by NIH HRP, Dr. James E.K. Hildreth, Lot/Batch number: 170136, Cat.-No.: ARP-723
NIH45-46 G54W Anti-HIV-1 gp120 mAb, provided by NIH HRP, Dr. Pamela Bjorkman32, Lot/Batch number: 160101, Cat.-No.: ARP-12174
VRC-CH31 Anti-HIV-1 Envelope CD4 Binding Site mAb, provided by NIH HRP, Drs. Barton F. Haynes and Hua-Xin Liao, Lot/Batch number: 140161, Cat.-No.: ARP-12565
Regdanvimab, Therapeutic anti-SARS-CoV-2-spike mAb, Celltrion, Lot/Batch-No.: 21006342
Tixagevimab (AZD8895), Prophylactic anti-SARS-CoV-2-spike mAb, AstraZeneca, Charge: CAAK
Cilgavimab (AZD1061), Prophylactic anti-SARS-CoV-2-spike mAb, AstraZeneca, Charge: CAAK
Avelumab (Bavencio), Therapeutic PD-L1 mAb, Merck KGaA, Lot/Batch-No.: AU026181
Atezolizumab (Tecentriq), Therapeutic PD-L1 mAb, Roche, Lot/Batch-No.: H0105
Nivolumab (Opdivo), Therapeutic PD-1 mAb, Bristol-Myers Squibb GmbH & Co, Lot/Batch-No.: AAT5725
Utomilumab (PF-05082566), Therapeutic 4-1BB agonistic mAb Creative Biolabs, Lot/Batch number: CB1900108, Cat.-No.: TAB-457CQ
Urelumab (BMS-663513), Therapeutic 4-1BB agonistic mAb Creative Biolabs, Lot/Batch number: CB0421JF01, Cat.-No.: TAB179
Human 4 1BB/TNFRSF9/CD137, Polyclonal antibodies Biotechne, Lot/Batch number: CCO0217041, Cat.-No.: AF838

Validation

Commercially available antibodies were validated by the manufacturers:

Commercial research antibodies:

Biolgegend:
https://www.biolegend.com/en-us/quality/quality-control
Company Statement for Flow Cytometry Reagents:
-Specificity testing of 1-3 target cell types with either single- or multi-color analysis (including positive and negative cell types).
-Once specificity is confirmed, each new lot must perform with similar intensity to the in-date reference lot. Brightness (MFI) is evaluated from both positive and negative populations.
-Each lot product is validated by QC testing with a series of titration dilutions.

GenScript:
Biotin-Strep-II-tag, clone 5A9F9, https://www.genscript.com/product/documents/down?doc_name=2107K043_A01737_COA.pdf&file=manu_workorder/work_order_detail_upload/2021/08/13/1628833431414_562.pdf

Miltenyi Biotec:
FITC-c-myc, clone SH1-26E7.1.3, https://assets.miltenyibiotec.com/coa/CoA_130-116-485_5200905664.pdf
PE-hGITRL, clone REA841, https://www.miltenyibiotec.com/DE-en/products/gitrl-antibody-anti-human-reafinity-rea841.html#conjugate=pe:size=30-tests-in-60-ul

Jackson ImmunoResearch:
PE-F(ab')2 goat anti mouse IgG (H+L), https://www.jacksonimmuno.com/lots/000000152883
PE-F(ab')2 goat anti rabbit IgG (H+L), https://www.jacksonimmuno.com/lots/000000160005

Thermo Fisher Scientific
PE-V5 Tag, clone TCM5, https://www.thermofisher.com/order/genome-database/dataSheetPdf?producttype=antibody&productsubtype=antibody_primary&productId=12-6796-42&version=351
SARS-CoV-2 Spike, clone ARC2373, https://www.thermofisher.com/order/genome-database/dataSheetPdf?producttype=antibody&productsubtype=antibody_primary&productId=MA5-36087&version=351

R&D Systems
hGITRL, clone 109101, https://www.rndsystems.com/cofas/human-gitr-ligand-tnfsf18-antibody-109101_mab6941
B7H4, clone 973816, https://www.rndsystems.com/cofas/human-b7-h4-antibody-973816_mab65761
hBTN3A1/2/3, clone 849203, https://resources.rndsystems.com/pdfs/datasheets/mab7136.pdf?v=20231029&_ga=2.260332485.937768935.1698624997-721380453.1698624997

BD Biosciences
PE-mGITRL, clone MIH44, https://www.bdbiosciences.com/en-us/products/reagents/flow-cytometry-reagents/research-reagents/single-color-antibodies-ruo/pe-rat-anti-mouse-gitr-ligand.563541

biotechne
Human 4 1BB/TNFRSF9/CD137, https://www.bio-techne.com/p/antibodies/human-4-1bb-tnfrsf9-cd137-antibody_af838

Creative Biolabs
Utomilumab (PF-05082566), https://www.creativebiolabs.net/Utomilumab-67709.htm
Urelumab (BMS-663513), https://www.creative-biolabs.com/Anti-Human%204-1BB%20Recombinant%20Antibody_662_10.htm

Commercial antibodies for clinical use:
Regdanvimab, Celltrion, https://www.ema.europa.eu/en/documents/referral/regdanvimab-treatment-covid-19-celltrion-covid-19-article-53-procedure-assessment-report_en.pdf
Evusheld (Tixagevimab + Cilgavimab), AstraZeneca https://www.ema.europa.eu/en/documents/product-information/evusheld-epar-product-information_en.pdf
Atezolizumab, Roche https://www.ema.europa.eu/en/documents/product-information/tecentriq-epar-product-information_en.pdf
Avelumab, Merck KGaA https://www.ema.europa.eu/en/documents/product-information/bavencio-epar-product-information_de.pdf
Nivolumab, Bristol-Myers Squibb GmbH & Co https://www.ema.europa.eu/en/documents/product-information/opdivo-epar-product-information_en.pdf
ORTHOCLONE OKT3, JANSSEN-CILAG, Johnson & Johnson, as no certificate of analysis was available, this antibody was validated by confirming upregulation of T cell activation markers on PBMCs after incubatation beads coated with this antibody

Non-commercial antibodies:

B7H3, clone 7517 Provided by Otto Majdic. The B7-H3 antibody 7517 was validated by confirming that it reacted with BW5147 cell expressing B7-H3 (but not with the parental BW5147 cell line.
References:
DOI: 10.4049/jimmunol.172.4.2352

NIH HIV Reagent Program:

SIM.2 Anti-human CD4 mAb, https://www.hivreagentprogram.org/Catalog/HRPMonoclonalAntibodies/ARP-723.aspx
References:
1. McCallus DE, Ugen KE, Sato AI, Williams WV, Weiner DB. Construction of a recombinant bacterial human CD4 expression system producing a bioactive CD4 molecule. Viral Immunol 5:163-172, 1992.
2. Oravecz T, Norcross MA. Costimulatory properties of the human CD4 molecule: enhancement of CD3-induced T cell activation by

human immunodeficiency virus type 1 through viral envelope glycoprotein gp120. AIDS Res Hum Retroviruses 9:945-955, 1993.

NIH45-46 G54W Anti-HIV-1 gp120 mAb, https://www.hivreagentprogram.org/Catalog/HRPMonoclonalAntibodies/ARP-12174.aspx
Reference:
Diskin, R., Scheid, J. F., Marcovecchio, P. M., Klein, F., Gao, H., Gnanapragasam, P. N. P., Abadir, A., Seaman, M. S., Nussenzweig, M. C. & Bjorkman, P. J. (2011) Increasing the Potency and Breadth of an Anti-HIV Antibody using Structure-Based Rational Design. Science 334, 1289-1293.

VRC-CH31 Anti-HIV-1 Envelope CD4 Binding Site mAb, https://www.hivreagentprogram.org/Catalog/HRPMonoclonalAntibodies/ARP-12565.aspx
Reference:
Bonsignori, M., Montefiori, D. C., Wu, X., Chen, X., Hwang, K. K., Tsao, C. Y., Kozink, D. M., Parks, R. J., Tomaras, G. D., Crump, J. A., Kapiga, S. H., Sam, N. E., Kwong, P. D., Kepler, T. B., Liao, H. X., Mascola, J. R. & Haynes, B. F. (2012). Two distinct broadly neutralizing antibody specificities of different clonal lineages in a single HIV-1-infected donor: implications for vaccine design. J Virol 86, 4688-92.

Eukaryotic cell lines

Policy information about cell lines and Sex and Gender in Research

Cell line source(s)

The following base cell lines were used in this study:
Base for biosensor cell lines: JE6.1 NFκB-eGFP (in-house production prior to the start of this study), JE6.1 TPR (in house production), JE6.1 TCR knockout Nur77-mKO2 (provided by BS and ML)
Base for stimulator cell lines: BW5147, K562, HEK293T (all available in-house prior to the start of this study)
All engineered cell lines (biosensor cell lines and stimulator cell lines) were produced in-house by lenti- or retroviral transduction and expression of the respective molecules.

Authentication

All cells were regularly assessed morphologically. Additionally, prior to every series of experiments cells were authenticated by establishing their markers and functional profile by flow cytometry.

Mycoplasma contamination

Cell lines were regularily tested for Mycoplasma contamination using a well-established reporter cell based assay. (Battin, C. et al. A human monocytic NF-κBfluorescent reporter cell line for detection of microbial contaminants in biological samples. (2017) doi:10.1371/journal.pone.0178220.) No experiments were performed with mycoplasma contaminated stimulator or biosensor cells.

Commonly misidentified lines
(See ICLAC register)

No commonly misidentified cell lines were used.

Plants

Seed stocks

Report on the source of all seed stocks or other plant material used. If applicable, state the seed stock centre and catalogue number. If plant specimens were collected from the field, describe the collection location, date and sampling procedures.

Novel plant genotypes

Describe the methods by which all novel plant genotypes were produced. This includes those generated by transgenic approaches, gene editing, chemical/radiation-based mutagenesis and hybridization. For transgenic lines, describe the transformation method, the number of independent lines analyzed and the generation upon which experiments were performed. For gene-edited lines, describe the editor used, the endogenous sequence targeted for editing, the targeting guide RNA sequence (if applicable) and how the editor was applied.

Authentication

Describe any authentication procedures for each seed stock used or novel genotype generated. Describe any experiments used to assess the effect of a mutation and, where applicable, how potential secondary effects (e.g. second site T-DNA insertions, mosiacism, off-target gene editing) were examined.

Flow Cytometry

Plots

Confirm that:

☒ The axis labels state the marker and fluorochrome used (e.g. CD4-FITC).

☒ The axis scales are clearly visible. Include numbers along axes only for bottom left plot of group (a 'group' is an analysis of identical markers).

☒ All plots are contour plots with outliers or pseudocolor plots.

☒ A numerical value for number of cells or percentage (with statistics) is provided.

Methodology

Sample preparation

Samples were transferred to microtiter tubes and washed with FACS buffer (1xPBS, 0,5%FCS, 0,05% Sodium azide). If TCS/BW cells were used as stimulator cells mCD45.2-APC antibody was added. When HEK293T cells were used as stimulator cells, biosensor cells were stained with a CD28-APC antibody. Next, samples were incubated for 20min at 4°C and washed in FACS buffer. Samples were then analyzed directly or stored at 4°C for later analysis on the same day.

Instrument	FACSCalibur, LSRFortessa (both BD Bioscience, NJ, USA), CytoFlexS (Beckman Coulter, CA, USA)
Software	CellQuest V6.0 (BDBioscience,NJ,USA), FACSDiva V9.0 (BDBioscience,NJ,USA), CytExpert V2.4 (Beckman Coulter, CA, USA)
Cell population abundance	Sample purity was repeatedly determined by staining for the relevant surface markers and reporter gene expression. Representative histograms are included within the figures.
Gating strategy	FSC/SSCgate was set to the live lymphocyte population. Next the gate was set on APC/RFP- cells (reporter cells). There was always a clear distinction between APC/RFP positive and negative populations. Exemplary figures are provided in the Supplementary Information. The Nur77 reporter cell line was identified by expression of mAmetrine.

☒ Tick this box to confirm that a figure exemplifying the gating strategy is provided in the Supplementary Information.
